# Terahertz time-domain ellipsometry with high precision for the evaluation of GaN crystals with carrier densities up to 10^20^ cm^−3^

**DOI:** 10.1038/s41598-021-97253-z

**Published:** 2021-09-15

**Authors:** Verdad C. Agulto, Toshiyuki Iwamoto, Hideaki Kitahara, Kazuhiro Toya, Valynn Katrine Mag-usara, Masayuki Imanishi, Yusuke Mori, Masashi Yoshimura, Makoto Nakajima

**Affiliations:** 1 Institute of Laser Engineering, Osaka University, Suita, Osaka 565-0871 Japan; 2 Nippo Precision Co., Ltd., Nirasaki, Yamanashi 407-0175 Japan; 3 Graduate School of Engineering, Osaka University, Suita, Osaka 565-0871 Japan

**Keywords:** Optical techniques, Characterization and analytical techniques, Materials for devices, Semiconductors

## Abstract

Gallium nitride (GaN) is one of the most technologically important semiconductors and a fundamental component in many optoelectronic and power devices. Low-resistivity GaN wafers are in demand and actively being developed to improve the performance of vertical GaN power devices necessary for high-voltage and high-frequency applications. For the development of GaN devices, nondestructive characterization of electrical properties particularly for carrier densities in the order of 10^19^ cm^−3^ or higher is highly favorable. In this study, we investigated GaN single crystals with different carrier densities of up to 10^20^ cm^−3^ using THz time-domain ellipsometry in reflection configuration. The *p*- and *s*-polarized THz waves reflected off the GaN samples are measured and then corrected based on the analysis of multiple waveforms measured with a rotating analyzer. We show that performing such analysis leads to a ten times higher precision than by merely measuring the polarization components. As a result, the carrier density and mobility parameters can be unambiguously determined even at high conductivities.

## Introduction

The profound interest in the GaN semiconductor is fueled by its superior properties—wide bandgap, high electron saturation velocity, high breakdown voltage, and high thermal conductivity. It has undoubtedly revolutionized today’s technology with the realization of blue light-emitting diodes (LEDs) and post-silicon power devices contributing to energy saving^1–3^. Moreover, it delivers performance and practicality for high-frequency and high-power device applications. Many III-nitride-based power electronic and optoelectronic devices require high free carrier concentrations, thus the motivation for doping investigations on GaN^4,5^. For next-generation power devices, the newly developed vertical GaN transistors, which show great potential for high-current and high-voltage operations, demand GaN wafers with high quality and low resistance^2,3,6,7^. In deep ultraviolet LEDs, GaN-based carrier injection layers with doping concentrations up to 10^19^ cm^−3^ are used^8,9^. There is also a need for tunable plasmonic sensors working in the mid-infrared region for on-chip detection of biomarkers, and doping GaN to achieve very high carrier concentrations up to 10^20^ cm^−3^ paves the way to meet such application^10^. With the ever-increasing demand for semiconductors with widely tunable carrier concentrations, there is an urgent and equal need for characterization techniques that are capable of measuring very high carrier concentrations. It is also highly favorable that such characterization is nondestructive for practicality. Terahertz (THz) spectroscopy is widely known to be advantageous and beneficial in this regard for its contact-free measurements, although it comes with limitations on the range of carrier concentrations that is measurable. This study demonstrates the applicability of THz waves to semiconductors with carrier densities up to the order of 10^20^ cm^−3^ and potentially higher using THz time-domain ellipsometry with high precision. We anticipate this technique to be widely useful in various fields dealing with highly doped semiconductors. GaN is also a promising semiconductor for the future of THz devices due to its aforementioned properties and its capability of providing high two-dimensional electron densities and high longitudinal phonon mode^11^. Moreover, 5G applications of GaN high-electron-mobility transistors (HEMT) have already penetrated the industrial market, and this material continues to be the leading candidate for 6G devices operating beyond 100 GHz. Therefore, continuous investigation of GaN in the THz frequency region is necessary.

In the last few decades, the use of THz radiation has significantly expanded across multidisciplinary fields and has traversed from once an emerging domain to now an established technology that opened new opportunities to access and study different physical phenomena. Owing to the low, non-ionizing energy of THz waves and their penetrative power through a variety of materials, THz science and technology finds a breadth of applications from bioimaging, noninvasive medical diagnostics, materials analysis and property modification, to industrial applications such as in-line monitoring, non-destructive testing, and defense and security imaging^12–40^. THz spectroscopy, in particular, allows the study of ultrafast and nonlinear phenomena, low-energy elementary excitations in condensed matter, and free-carrier transport properties^19–40^. One of the widely used techniques is the THz time-domain spectroscopy (THz-TDS) which determines a material’s complex refractive index by sampling the THz electric field in the time domain and monitoring the change in its amplitude and phase after interacting with the material via transmission or reflection. Since THz waves are sensitive to charge carrier dynamics, THz-TDS is widely used in semiconductors research to probe free-carrier transport properties. However, THz-TDS in transmission and reflection geometries present some difficulties and limitations. THz-TDS requires reference measurements, either through an aperture or substrate for transmission and standard mirror for reflection. Transmission-type THz-TDS is limited by the sample thickness and is not suitable for optically dense materials with significant absorption and high energy loss such as highly doped semiconductors. The carrier concentration range typically demonstrated in transmission THz-TDS is 10^14^–10^16^ cm^−3^ for few mm-thick wafers^30,31^. For thin films, carrier concentrations up to 10^18^ cm^−3^ can be measured for thicknesses of a few microns^32–34^ and higher if the thickness is in the nanometer scale^35–37^. Although reflection-type THz-TDS^38–40^ is available for thick, highly absorbing samples, sub-micron precision in the relative positions of the sample and the reference mirror is crucial; otherwise, the resulting phase error leads to inaccurate optical constants.

In comparison to transmission or reflection THz-TDS, the key feature of THz time-domain ellipsometry is it dispenses with the need to measure the incident THz wave to be able to evaluate the optical constants of the material under investigation. THz time-domain ellipsometry characterizes the *p*- and *s*-polarized THz waves upon reflection off a sample^41,42^. Linearly polarized THz waves are incident on the sample at the Brewster’s angle (or termed as pseudo-Brewster’s angle for absorbing materials) where the difference in reflection coefficients of *p*- and *s*-polarizations is maximized. From the amplitude ratio and phase difference (called the ellipsometric parameters) of the measured *p*- and *s*-polarized waves, the optical constants and film thickness of the sample can be determined. Therefore, THz time-domain ellipsometry is an advantageous nondestructive tool to probe free-carrier properties of GaN and other semiconductors without having to do separate measurements with and without the sample or with a bare substrate for thin film characterization. THz ellipsometry can also be performed via frequency-domain measurements^43,44^. With the use of a continuous wave source, THz frequency-domain ellipsometry is a purely intensity-based measurement; hence, the ellipsometric parameters are obtained by nonlinear regression analysis of the experimental data. In contrast, THz time-domain ellipsometry measurements detect both intensity and phase information, thereby allowing for the direct measurement of the ellipsometric parameters. Additionally, the spectral range of THz frequency-domain measurements typically cover below 1 THz because of the limitation in available continuous wave THz sources^45^. In characterizing semiconductors with very high carrier densities that have abrupt refractive index dispersion at very low frequencies, observing the optical response at a wider frequency range is desirable to obtain a more accurate set of free-carrier parameters. The developments and applications of THz ellipsometry are reviewed in detail in Refs.^46,47^.

THz ellipsometry technology has also been combined with external magnetic fields in a phenomenon called the optical Hall effect (OHE), which is analogous to the electric Hall effect but occurring at high (i.e., optical) frequencies. This method (also called magneto-optical ellipsometry) allows for the determination of the effective mass and type (*p* or *n*) of free carriers independently in addition to the carrier density and mobility^48^. THz-OHE measurements have been used to characterize Si homojunctions, AlGaN/GaN heterostructures, epitaxial graphene, and very low-doped semiconductors^49–53^. A numerical study on the magneto-optical Kerr effect of GaN in the THz region under different carrier densities and external magnetic fields has also been conducted^54^. Although THz magneto-optical ellipsometry can provide additional information on free-carrier properties, the use of magnets might not be convenient experimentally. In this case when the application of external magnetic fields is not possible, the effective mass and type of free carriers in the material under investigation are assumed to be known. In the case of the GaN semiconductor, the effective mass is already accurately known^55^ and is found to be constant for a wide range of carrier densities^56^. Hence, THz time-domain ellipsometry without the use of magnets is sufficiently powerful, while being more compact, to evaluate the carrier density and mobility of GaN crystals, especially those with very high carrier densities.

Previous THz time-domain ellipsometry studies on other relevant semiconductors such as Si wafer and GaAs thin films demonstrated the measurement with ~ 10^18^ cm^−3^ carrier densities^42,57^. In a recent study which investigated a GaN wafer and epitaxial layer using THz time-domain ellipsometry, the carrier densities are also in the 10^18^ cm^−3^ order^58^. Compared to previous reports, our study focuses on the evaluation of higher carrier densities up to 10^20^ cm^−3^ and demonstrates the accuracy of THz time-domain ellipsometry in evaluating very high conductivities. The use of THz time-domain ellipsometry for the measurement of an InAs wafer and ITO thin film with ~ 10^17^ cm^−3^ and ~ 10^20^ cm^−3^ carrier concentrations, respectively, has been reported^59^. Whereas the previous study utilized ultra-broadband emission up to 30 THz, our ellipsometry system employs a 1-THz source to characterize GaN crystals of similarly high carrier concentrations. Additionally, we show herein the potential of our THz time-domain ellipsometry system to accurately characterize even higher carrier densities up to 10^21^ cm^−3^. As such, THz time-domain ellipsometry extends the maximum range of carrier densities that can be reasonably measured by THz-TDS techniques with sufficient accuracy.

## Methods

Two free-standing GaN single crystals with different concentrations of oxygen impurities were investigated in this study. The first GaN sample (LPE-GaN) was grown on a GaN template using liquid phase epitaxy and has ~ 10^17^ cm^−3^ oxygen concentration^60,61^. The second sample (PS-GaN) is a point-seed crystal grown via the Na-flux method and has ~ 10^20^ cm^−3^ oxygen concentration^62,63^. Both samples are *c*-oriented. The details of the crystal growth are discussed in the abovementioned references. Based on Hall measurements, the LPE-GaN sample has a carrier density of 8.8 × 10^17^ cm^−3^, mobility of 296 cm^2^/Vs, and dc resistivity of 2.4 × 10^–2^ Ωcm, whereas the PS-GaN sample has a carrier density of 1.6 × 10^20^ cm^−3^, mobility of 44 cm^2^/Vs, and dc resistivity of 8.6 × 10^–4^ Ωcm.

**Figure 1:**
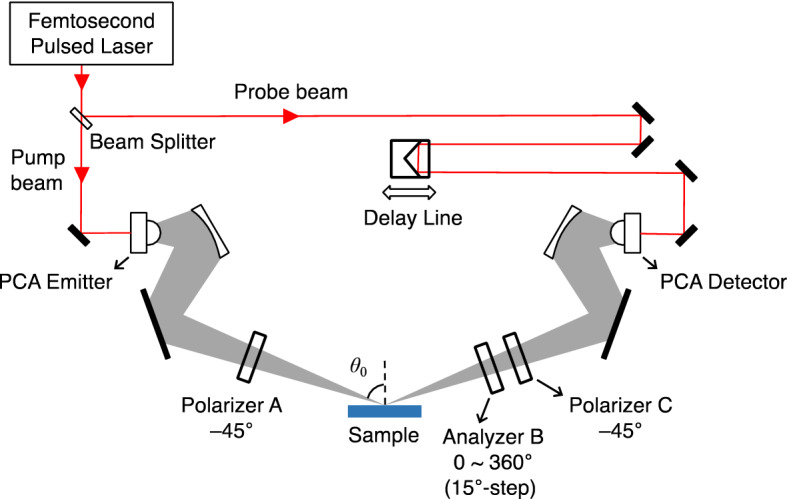
Schematic diagram of the THz time-domain ellipsometry setup.

**Figure 2:**
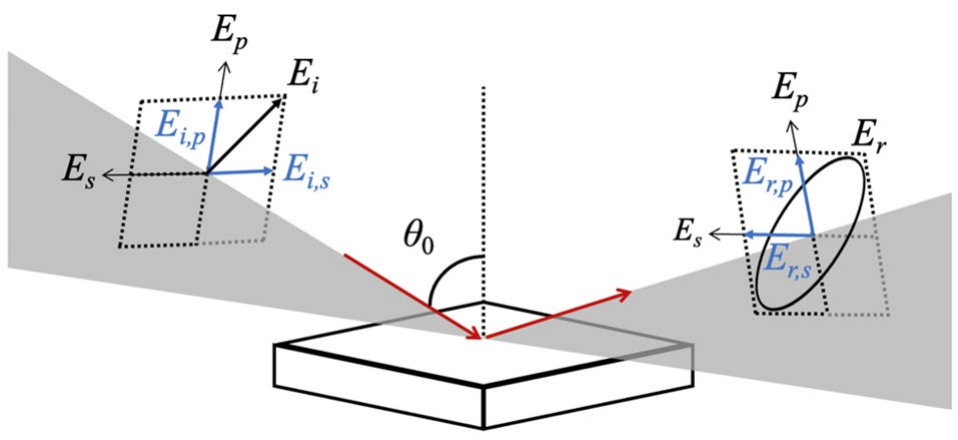
Propagation and polarization directions of the incident (*E*_*i*_) and reflected (*E*_*r*_) THz waves. *E*_*p*_ and *E*_*s*_ denote the directions of the *p*- and *s*-polarizations corresponding to 0° and 90°, respectively. *E*_*i*_ is linearly polarized at − 45°. Upon reflection, *E*_*r*_ becomes elliptically polarized owing to the difference in the *p*- and *s*- reflection coefficients.

THz time-domain ellipsometry was performed using Tera Evaluator, which is developed by Nippo Precision, Co., Ltd. in collaboration with our group. Figure 1 shows the schematic diagram of the THz ellipsometer. The setup consists of a femtosecond laser (pulse width < 120 fs; center wavelength ~ 800 nm) that is used to excite low-temperature-grown GaAs (LT-GaAs) dipole-type photoconductive antennas (PCA) for THz generation and detection. The generated THz waves from the PCA emitter pass through Polarizer A that is rotated at an angle of *θ*_*A*_ = − 45° to ensure the *p*- and *s*-polarization components have equal intensities. Since the photoconductive antenna is sensitive to THz polarization, the angular position of Polarizer C before the PCA detector is also set at *θ*_*C*_ = − 45°. The PCAs are also oriented at − 45°, thus, the polarizers do not diminish the THz signals but are rather placed to minimize errors. The angular positions are defined based on the coordinate system shown in Fig. 2 which illustrates the propagation and polarization directions of the incident and reflected THz waves. The THz waves are incident on the sample surface at an angle *θ*_0_ = 70°, and the reflected waves are measured at different angular positions of Analyzer B from *θ*_*B*_ = 0° to 360° at 15° step, wherein the 0° and 90° angular positions correspond to the *p*- and *s*-polarizations, respectively.

The multiple analyzer angle measurements are performed to eliminate systematic errors from the signals. The extraction of the desired *p*- and *s*-polarization components is as follows. Let *E*_*A*_ be the signal that has passed through Polarizer A which is oriented at an angle *θ*_*A*_. The amplitudes of *p*- and *s*-polarized THz waves incident on the sample are then given by ${E}_{sample,p}={E}_{A}\cos{\theta }_{A}$ and ${E}_{sample,s}={E}_{A}\sin{\theta }_{A}$, respectively. The reflected signal just after the sample can then be represented as ${E}_{A,p}={r}_{p}{E}_{A}\cos{\theta }_{A}$ and ${E}_{A,s}={r}_{s}{E}_{A}\sin{\theta }_{A}$ where *r*_*p*_ and *r*_*s*_ are the sample’s reflection coefficients. At any given analyzer angle *θ*_*B*_ the reflected signal that passes through the analyzer can be expressed as ${E}_{B}={E}_{A,p}\cos{\theta }_{B}+{E}_{A,s}\sin{\theta }_{B}$. Then, the signal that passes through Polarizer C can be expressed as ${E}_{C}={E}_{B}\cos\left({\theta }_{B}-{\theta }_{C}\right)$ . Evaluating these relations leads to the following expression for *E*_*C*_:

1 $${E}_{C}=A \cos 2{\theta }_{B}+B \sin 2{\theta }_{B}+C,$$

where $A=\frac{1}{2}\left[{E}_{A,p} \cos{\theta }_{C}-{E}_{A,s}\sin{\theta }_{C}\right]$, $B=\frac{1}{2}\left[{E}_{A,p}\sin{\theta }_{C}+{E}_{A,s}\cos{\theta }_{C}\right]$, and $C=\frac{1}{2}\left[{E}_{A,p}\cos{\theta }_{C}+{E}_{A,s}\sin{\theta }_{C}\right]$. At each *θ*_*B*_ position, the reflected time-domain waveform is measured. For each point of the time-domain waveform, Eq. (1) describes the detected amplitude depending on the *θ*_*B*_ orientation. Taking all the measured time-domain waveforms after a full rotation of *θ*_*B*_, the values of *A*, *B*, and *C* at each time are determined by fitting the detected amplitudes for various *θ*_*B*_ positions to Eq. (1). At any certain time, the deviation of the measured amplitude from the idealized trend represented by Eq. (1) can be attributed to data acquisition timing jitter. In such case, time-shift is performed accordingly on the entire waveform to compensate for the jitter. Consequently, the phase is also corrected in the process. This correction follows the assumption that the shape of all waveforms is similar, thus the amplitude error is less than the phase error which is reasonable as the polarizers have an extinction ratio of ~ 10^–5^. Based on Eq. (1), the corrected waveforms of the reflected *p*- and *s*-polarizations (i.e., at *θ*_*B*_ = 0° and *θ*_*B*_ = 90°, respectively) are extracted as ${E}_{p}=A+C=\frac{{r}_{p}{E}_{A}}{2}$ and ${E}_{s}=-A+C=\frac{{r}_{s}{E}_{A}}{2}$ for *θ*_*A*_ = *θ*_*C*_ = − 45°. This analysis method corrects the amplitude and phase components of the THz waveforms which is very useful for time-domain ellipsometry and which makes it unique from the continuous-wave ellipsometry with rotating analyzer. As such, the multiple-angle measurements can eliminate the contribution of systematic errors to the measured *p*- and *s*- time-domain waveforms, hence a high accuracy in ellipsometric parameters. The standard deviation of the ellipsometric parameters is less than 0.0025. As a result, the refractive index can be determined with good accuracy and precision.

## Results and discussion

**Figure 3:**
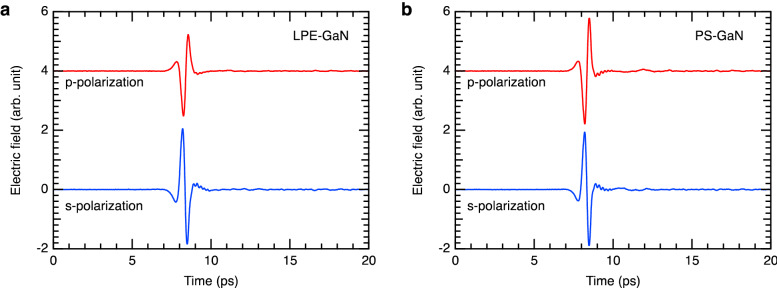
Time-domain waveforms of the *p*- and *s*-polarizations reflected from the GaN samples.

Figure 3 shows the experimental time-domain waveforms of the reflected *p*- and *s*-polarizations measured from the LPE-GaN and PS-GaN samples. The amplitude of *p*-polarization is higher for the PS-GaN sample than the LPE-GaN sample, which can be explained by the higher absorption in the PS-GaN sample due to the larger number of free carriers. In general, the reflectance of *p*-polarization decreases with increasing incidence angle and disappears at the Brewster angle in the case of non-absorbing materials. If there is absorption in the medium, reflection of *p*-polarization occurs at the pseudo-Brewster angle. A higher reflectance of *p*-polarization thereby indicates higher absorption. The amplitude of *s*-polarization, on the other hand, is similar for the two samples since *s*-polarization has low sensitivity to absorption.

**Figure 4:**
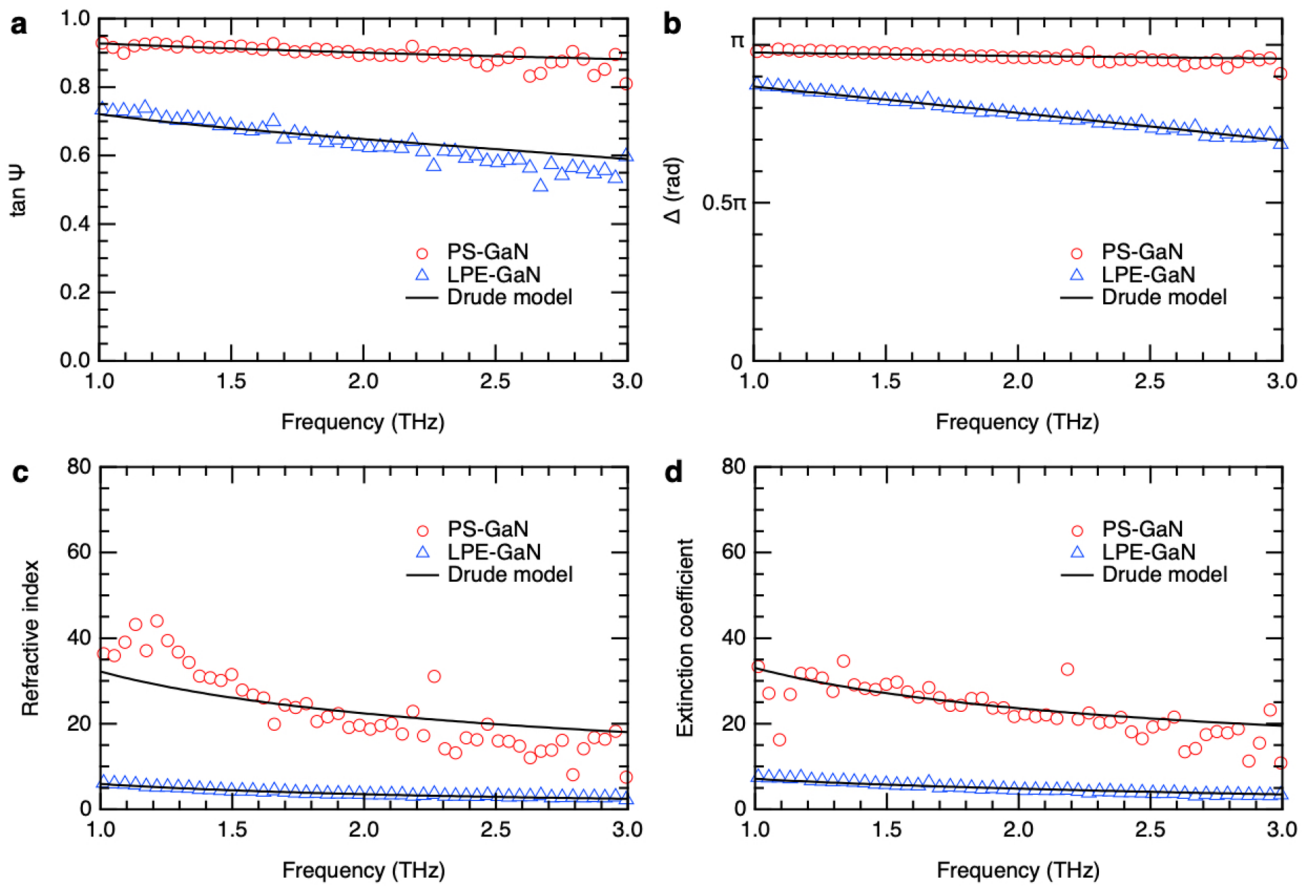
Ellipsometric parameters: (**a**) Amplitude ratio and (**b**) phase difference of the reflected *p*- and *s*-polarizations. (**c**) Refractive index and (**d**) extinction coefficient spectra of the GaN samples.

The time-domain waveforms are Fourier-transformed to obtain the ellipsometric parameters, which are defined from the ratio ($\stackrel{\sim }{\rho }$) of the complex amplitude reflection coefficients for *p*- and *s*-polarizations, $\tilde{r }_{p}$ and $\tilde{r }_{s}$^41^:

2 $$\stackrel{\sim }{\rho } \equiv {\tan \Psi \exp}\left(i\Delta \right)\equiv \frac{\tilde{r }_{p}}{{\tilde{r }}_{s}}\equiv \left(\frac{\tilde{E }_{r,p}}{\tilde{E }_{i,p}}\right)/\left(\frac{\tilde{E }_{r,s}}{\tilde{E }_{i,s}}\right),$$

where tanΨ is the amplitude ratio and Δ is the phase difference. $\tilde{E }_{r,p}$ and $\tilde{E }_{r,s}$ are the polarization components reflected off the sample, whereas $\tilde{E }_{i,p}$ and $\tilde{E }_{i,s}$ are the incident polarizations. Since the incident THz radiation is linearly polarized at − 45° relative to the plane of incidence, the incident polarization components are equal, i.e., $\tilde{E }_{i,p}=\tilde{E }_{i,s}$. The ellipsometric parameters obtained for the LPE-GaN and PS-GaN samples in the 1 –3 THz frequency range are shown in Fig. 4a,b. As the carrier density increases, the value of tanΨ approaches 1 whereas the value of Δ approaches π. The precision of the THz ellipsometry measurements is thus crucial in evaluating high carrier densities as the dispersion of the ellipsometric parameters becomes narrow.

Wurtzite GaN is a uniaxial material, i.e., the refractive index along the *a*-axis and *b*-axis are the same and different along the *c*-axis. Our GaN samples are *c*-oriented and thus have an isotropic surface, and multiple reflections inside the material are not involved. Therefore, we can use the Fresnel equations for reflection off an air/sample interface assuming an isotropic sample which are given by^41^:

3 $$\tilde{r }_{p}=\frac{\tilde{n }_{1}\cos{\theta }_{0}-\tilde{n }_{0}\cos{\theta }_{1}}{\tilde{n }_{1}\cos{\theta }_{0}+\tilde{n }_{0}\cos{\theta }_{1}};$$

$$\tilde{r }_{s}=\frac{\tilde{n }_{0}\cos{\theta }_{0}-\tilde{n }_{1}\cos{\theta }_{1}}{\tilde{n }_{0}\cos{\theta }_{0}+\tilde{n }_{1}\cos{\theta }_{1}},$$

where $\tilde{n }_{1}$ and $\tilde{n }_{0}$ are the complex refractive indices of the sample and the atmosphere (air), respectively, and *θ*_1_ is the angle of refraction. The relation of the refractive index to the dielectric function ($\stackrel{\sim }{\varepsilon }$) is given by ${\stackrel{\sim }{\varepsilon }=\tilde{n }}^{2}$. The pseudo-dielectric function^41^ (i.e., assuming a flat surface and semi-infinite thickness) of the bulk sample can be directly calculated from the ellipsometric parameters. By evaluating the Fresnel equations, the complex refractive index of the GaN samples can then be deduced from the ellipsometric parameters using the equation^41^:

4 $${\stackrel{\sim }{\varepsilon }=\left({n}_{1}-i{\kappa }_{1}\right)}^{2}={\tilde{n }}_{0}^{2} {\sin}^{2}{\theta }_{0}\left[{\left(\frac{1-\stackrel{\sim }{\rho }}{1+\stackrel{\sim }{\rho }}\right)}^{2}\times {\mathrm{tan}}^{2}{\theta }_{0}+1\right],$$

where *n*_1_ and *κ*_1_ are the real (refractive index) and imaginary (extinction coefficient) parts of the complex refractive index, respectively. The complex refractive index spectra of the GaN samples are shown in Fig. 4c,d. The increasing trend of the refractive index and extinction coefficient with decreasing frequency is attributed to free carriers. The PS-GaN sample has a higher concentration of free carriers, thus it exhibits higher complex refractive index. Furthermore, phonon absorption is not observed since the lowest phonon mode of GaN is ~ 16 THz^55^ which is far from our measurement region. Therefore, the complex refractive index can be analyzed using the Drude model given by the equation,

5 $${\tilde{n }}^{2}\left(\omega \right)={\varepsilon }_{s}-\frac{{e}^{2}N}{{\varepsilon }_{0}{m}^{*}\left({\omega }^{2}-i\omega /\tau \right)},$$

where *ω* is angular frequency, *ε*_*s*_ is the static dielectric constant, *e* is electron charge, *N* is carrier density, *ε*_0_ is free space permittivity, m^*^ is effective mass, and *τ* is scattering time, which is related to the mobility (*μ*) by $\mu =e\tau /{m}^{*}$ . The static dielectric constant of GaN and effective mass were taken to be *ε*_*s*_ = 9.22 and m^*^ = 0.237*m*_*e*_ (where *m*_*e*_ is free electron mass)^55,64^, whereas the carrier density and mobility were treated as fitting parameters. The refractive index and extinction coefficient spectra were simultaneously fitted. The best-fit parameters are *N* = 9.3 × 10^17^ cm^−3^ and *μ* = 345 cm^2^/Vs in the case of the LPE-GaN sample, and *N* = 2.1 × 10^20^ cm^−3^ and *μ* = 36 cm^2^/Vs in the case of the PS-GaN sample, and their dc resistivities are 1.9 × 10^–2^ Ωcm and 8.4 × 10^–4^ Ωcm, respectively. These parameters are used to model the ellipsometric parameters as well. For the PS-GaN sample, the discrepancy of the experimental refractive index from the Drude model below 1.5 THz is attributed to the smaller dimensions of the sample compared to the THz beam spot. The experimental results below 1 THz are not shown for this reason. Nevertheless, the THz ellipsometry results analyzed using the Drude model match the electrical characterization results of the Hall measurements. It is also noteworthy that using THz ellipsometry we have demonstrated the accurate evaluation of carrier densities in the order of 10^20^ cm^−3^ which is 2 to 3 orders of magnitude higher than the typical measurement range of THz-TDS.

**Figure 5:**
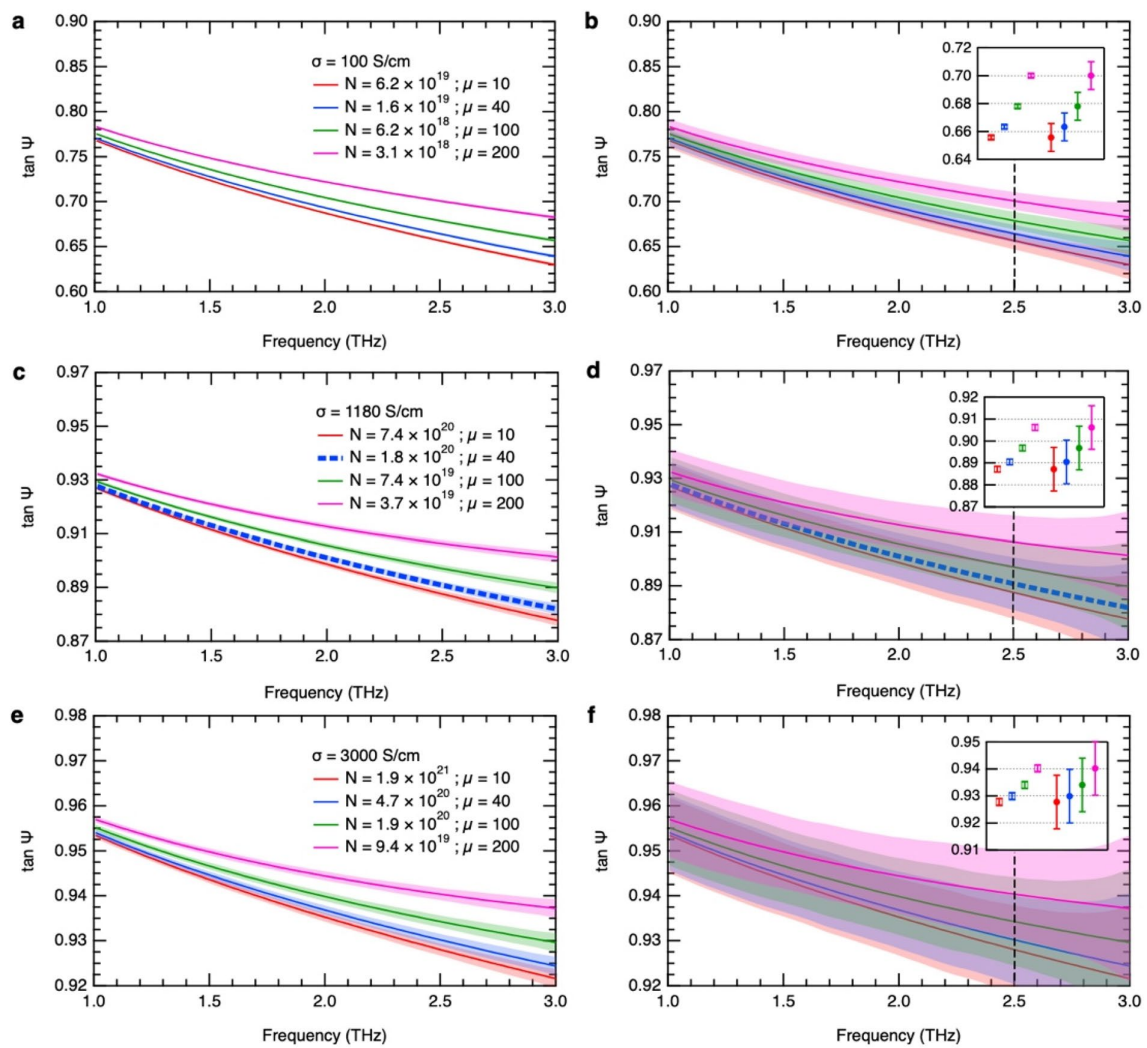
Calculated amplitude ratio for different conductivities: 100, 1180, and 3000 S/cm with varying carrier density and mobility. The dashed blue line corresponds to material parameters close to the PS-GaN sample. The shaded regions represent the uncertainties of THz time-domain ellipsometry with multiple-angle measurements (**a**,**c**,**e**) and without, i.e., measurements at 0° and 90° polarizer angles only (**b**,**d**,**f**). For easier comparison, the respective values at 2.5 THz are shown by the insets (hollow squares—with multiple-angle measurements; solid circles—0° and 90° measurements only).

**Figure 6:**
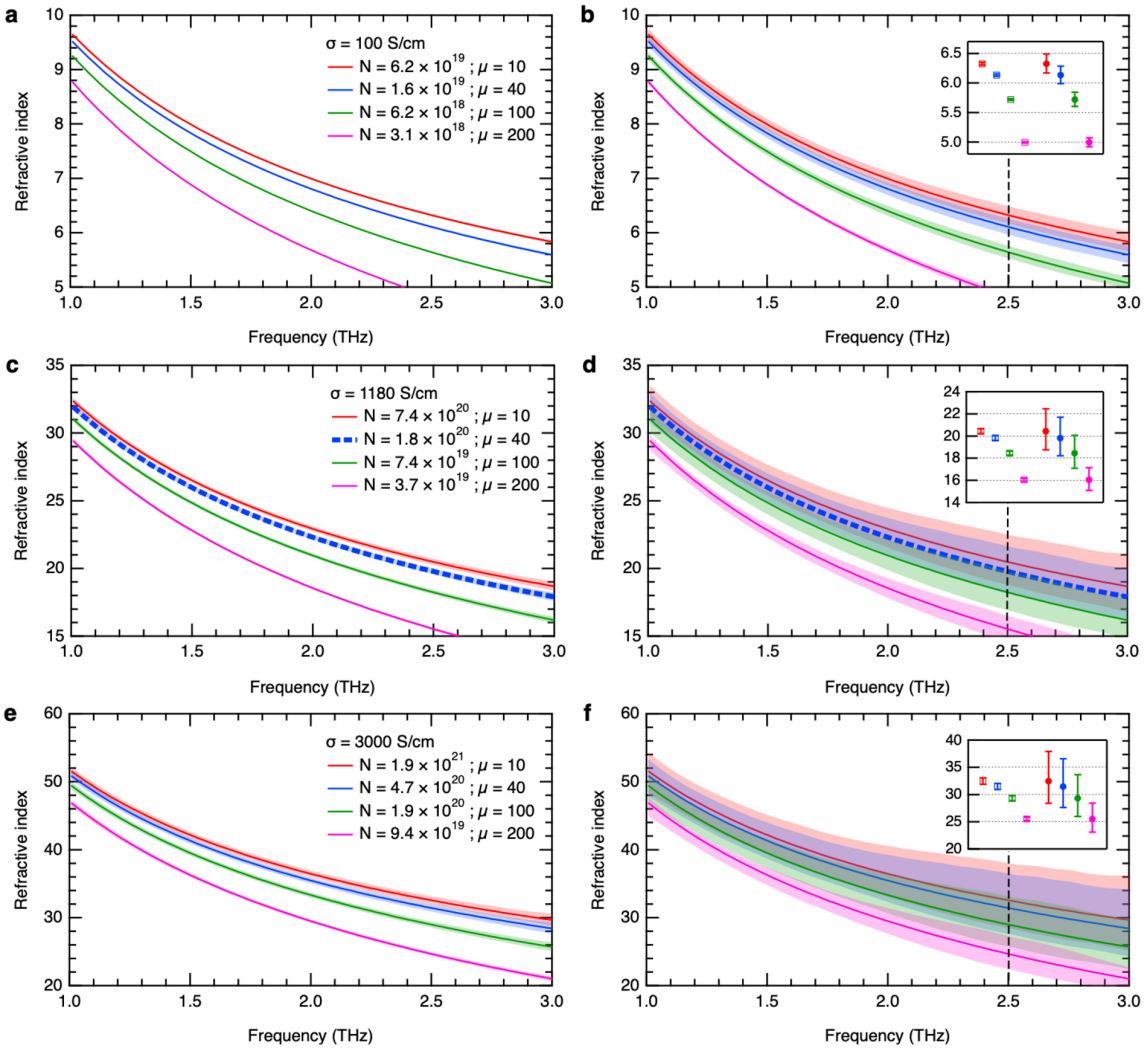
Calculated refractive index spectra for different conductivities: 100, 1180, and 3000 S/cm with varying carrier density and mobility. The dashed blue line corresponds to material parameters close to the PS-GaN sample. The shaded regions represent the uncertainties of THz time-domain ellipsometry with multiple-angle measurements (**a**,**c**,**e**) and without, i.e., measurements at 0° and 90° polarizer angles only (**b**,**d**,**f**). For easier comparison, the respective values at 2.5 THz are shown by the insets (hollow squares—with multiple-angle measurements; solid circles—0° and 90° measurements only).

From the optical constants the dc conductivity or the dc resistivity is relatively easy to determine accurately. However, for a particular conductivity, it is rather difficult to distinguish the correct values of carrier density and mobility especially at high carrier concentrations. Thus, we further demonstrate the accuracy of our THz ellipsometry system with multiple-angle measurements in distinguishing between different values of carrier density and mobility. Figures 5 and 6 show the calculated amplitude ratio and refractive index spectra, respectively, for different conductivities and possible carrier density and mobility values for each conductivity based on the Drude model. In Fig. 5, the amplitude ratio increases with increasing mobility for a constant conductivity. This behavior is because of the increase in the imaginary part of the complex refractive index. The absorption is higher due to the longer scattering time, which is proportional to the mobility. The reflectivity then increases due to the higher free-carrier absorption. It can be consistently seen in each graph in Figs. 5 and 6 that as the carrier concentration becomes higher, the difference in the curves becomes more subtle. Consequently, ambiguity arises in determining the accurate carrier density and mobility parameters. Herein we show the advantage of our technique utilizing multiple-angle measurements over measuring only at 0° and 90° to determine the *p*- and *s*-polarizations, by comparing the uncertainties in these two types of measurement. We experimentally confirmed that the standard deviation of ellipsometric parameters extracted from multiple-angle measurements is about ten times less than that of directly measured *p*- and *s*-polarized waves for the same total measurement time. The shaded regions in Fig. 5 represent the measured standard deviation of ellipsometric parameters obtained with multiple-angle measurements (a, c, e) and without (b, d, f). The resulting error in refractive index, which also includes the phase difference error, is then estimated and represented by the shaded regions in Fig. 6. Comparing the relative errors, the refractive index has a lower relative uncertainty because of its higher magnitude. Three cases are demonstrated: 1) low conductivity of σ = 100 S/cm; 2) σ = 1180 S/cm which is close to that of the PS-GaN sample; and 3) high conductivity of σ = 3000 S/cm. For the low conductivity case (Fig. 6a,b), each refractive index dispersion is easily distinguishable by the two methods. For the conductivity close to that of our PS-GaN sample (Fig. 6c,d), our technique can still clearly distinguish between different carrier densities in the order of 10^20^ cm^−3^ whereas the other method already has significant uncertainty. For the high conductivity case (Fig. 6e,f), the results suggest that our technique can potentially be used to evaluate carrier densities up to the order of 10^21^ cm^−3^. For easier comparison, the insets in Figs. 5 and 6 show the respective values at 2.5 THz with the uncertainty of our system and the direct measurement approach. The error bars clearly show that the technique using multiple-angle measurements is superior in discriminating between small differences in ellipsometric parameters and refractive index. These results show that as conductivity increases, the accuracy of the measurements becomes more crucial in parameter estimation. Since the THz ellipsometry with multiple-angle measurements allows for amplitude and phase corrections of the measured time-domain waveforms, the experimental ellipsometric parameters have high accuracy. As a result, we can evaluate the carrier density and mobility uniquely even for high conductivities. Therefore, our technique extends the range of carrier concentrations measurable by THz-TDS and significantly improves the precision of THz time-domain ellipsometry for the characterization of very high carrier densities.

## Conclusion

Using THz time-domain ellipsometry, we demonstrated the investigation of GaN bulk semiconductors with high carrier densities of up to 10^20^ cm^−3^ and potentially higher. By extracting the *p*- and *s*-polarizations from a set of measurements at different analyzer angles instead of direct detection of these components, highly precise ellipsometric parameters can be obtained. As a result, the unambiguous evaluation of carrier density and mobility even at high conductivities is possible. Thus, our system extends the usability of THz spectroscopy for the characterization of thick, optically dense semiconductors. It is also applicable to thin films owing to the penetrative power of THz waves. Moreover, it is a nondestructive, contactless, and reference-free measurement technique. Therefore, high-precision THz time-domain ellipsometry is a superior characterization method for semiconductors and other materials with high carrier concentrations toward the development of future devices.

## References

[1] Amano H (2015). Growth of GaN on sapphire via low-temperature deposited buffer layer and realization of p-type GaN by Mg doping followed by low-energy electron beam irradiation (Nobel Lecture). Ann. Phys..

[2] Takino J (2019). Development of a 2-inch GaN wafer by using the oxide vapor phase epitaxy method. Jpn. J. Appl. Phys..

[3] Kachi T (2014). Recent progress of GaN power devices for automotive applications. Jpn. J. Appl. Phys..

[4] Fritze S (2012). High Si and Ge n-type doping of GaN doping—Limits and impact on stress. Appl. Phys. Lett..

[5] Sarkar B (2017). High free carrier concentration in p-GaN grown on AlN substrates. Appl. Phys. Lett..

[6] Takino J (2020). Extreme reduction of on-resistance in vertical GaN p–n diodes by low dislocation density and high carrier concentration GaN wafers fabricated using oxide vapor phase epitaxy method. Appl. Phys. Express.

[7] Ueda T (2019). GaN power devices: Current status and future challenges. Jpn. J. Appl. Phys..

[8] Jamil T, Usman M, Malik S, Jamal H (2021). The marvelous optical performance of AlGaN-based deep ultraviolet light-emitting diodes with AlInGaN-based last quantum barrier and step electron blocking layer. Appl. Phys. A.

[9] Collazo R (2011). Progress on n-type doping of AlGaN alloys on AlN single crystal substrates for UV optoelectronic applications. Phys. Status Solidi.

[10] Kirste R (2013). Ge doped GaN with controllable high carrier concentration for plasmonic applications. Appl. Phys. Lett..

[11] Ahi K (2017). Review of GaN-based devices for terahertz operation. Opt. Eng..

[12] Dhillon SS (2017). The 2017 terahertz science and technology roadmap. J. Phys. D. Appl. Phys..

[13] Mittleman DM (2017). Perspective: Terahertz science and technology. J. Appl. Phys..

[14] Kurihara T (2020). Reconfiguration of magnetic domain structures of ErFeO_3_ by intense terahertz free electron laser pulses. Sci. Rep..

[15] Li D (2019). Terahertz radiation from combined metallic slit arrays. Sci. Rep..

[16] Takano K (2019). Terahertz emission from gold nanorods irradiated by ultrashort laser pulses of different wavelengths. Sci. Rep..

[17] Makino K (2018). Significant volume expansion as a precursor to ablation and micropattern formation in phase change material induced by intense terahertz pulses. Sci. Rep..

[18] Tadokoro Y (2015). Measurement of beam profiles by terahertz sensor card with cholesteric liquid crystals. Opt. Lett..

[19] Agulto VC (2021). Anisotropic complex refractive index of β-Ga_2_O_3_ bulk and epilayer evaluated by terahertz time-domain spectroscopy. Appl. Phys. Lett..

[20] Nakajima M, Namai A, Ohkoshi S, Suemoto T (2010). Ultrafast time domain demonstration of bulk magnetization precession at zero magnetic field ferromagnetic resonance induced by terahertz magnetic field. Opt. Express.

[21] Yamaguchi K, Nakajima M, Suemoto T (2010). Coherent control of spin precession motion with impulsive magnetic fields of half-cycle terahertz radiation. Phys. Rev. Lett..

[22] Yamaguchi K, Kurihara T, Watanabe H, Nakajima M, Suemoto T (2015). Dynamics of photoinduced change of magnetoanisotropy parameter in orthoferrites probed with terahertz excited coherent spin precession. Phys. Rev. B.

[23] Nakajima M, Takubo N, Hiroi Z, Ueda Y, Suemoto T (2008). Photoinduced metallic state in VO_2_ proved by the terahertz pump-probe spectroscopy. Appl. Phys. Lett..

[24] Nakajima M, Takubo N, Hiroi Z, Ueda Y, Suemoto T (2009). Study of photo-induced phenomena in VO_2_ by terahertz pump-probe spectroscopy. J. Lumin..

[25] Nakajima M (2016). Application of terahertz field enhancement effect in metal microstructures. J. Infrared Millimeter Terahertz Waves.

[26] Kurihara T (2018). Macroscopic magnetization control by symmetry breaking of photoinduced spin reorientation with intense terahertz magnetic near field. Phys. Rev. Lett..

[27] Ohkoshi S (2019). Rapid Faraday rotation on ε-iron oxide magnetic nanoparticles by visible and terahertz pulsed light. J. Am. Chem. Soc..

[28] Ohkoshi S (2020). Magnetic-pole flip by millimeter wave. Adv. Mater..

[29] Fitzky, G., Nakajima, M., Koike, Y., Leitenstorfer, A. & Kurihara, T. Ultrafast control of magnetic anisotropy by resonant excitation of 4*f* electrons and phonons in Sm_0.7_Er_0.3_FeO_3_. *Phys. Rev. Lett.* **127**, 107401 (2021). 10.1103/PhysRevLett.127.107401 34533346

[30] Jeon T-I, Grischkowsky D (1997). Nature of conduction in doped silicon. Phys. Rev. Lett..

[31] van Exter M, Grischkowsky D (1990). Carrier dynamics of electrons and holes in moderately doped silicon. Phys. Rev. B.

[32] Huggard PG (2000). Drude conductivity of highly doped GaAs at terahertz frequencies. J. Appl. Phys..

[33] Lloyd-Hughes J, Jeon T-I (2012). A review of the terahertz conductivity of bulk and nano-materials. J. Infrared Millimeter Terahertz Waves.

[34] Guo HC, Zhang XH, Liu W, Yong AM, Tang SH (2009). Terahertz carrier dynamics and dielectric properties of GaN epilayers with different carrier concentrations. J. Appl. Phys..

[35] Tang J (2014). Determination of carrier concentration dependent electron effective mass and scattering time of n-ZnO thin film by terahertz time domain spectroscopy. J. Appl. Phys..

[36] Arezoomandan S (2018). THz characterization and demonstration of visible-transparent/terahertz-functional electromagnetic structures in ultra-conductive La-doped BaSnO_3_ Films. Sci. Rep..

[37] Ma G (2008). Carrier concentration dependence of terahertz transmission on conducting ZnO films. Appl. Phys. Lett..

[38] Howells SC, Schlie LA (1996). Transient terahertz reflection spectroscopy of undoped InSb from 0.1 to 1.1 THz. Appl. Phys. Lett..

[39] Pashkin A, Kempa M, Němec H, Kadlec F, Kužel P (2003). Phase-sensitive time-domain terahertz reflection spectroscopy. Rev. Sci. Instrum..

[40] Jeon T-I, Grischkowsky D (1998). Characterization of optically dense, doped semiconductors by reflection THz time domain spectroscopy. Appl. Phys. Lett..

[41] Fujiwara H (2007). Spectroscopic Ellipsometry: Principles and Applications.

[42] Nagashima T, Hangyo M (2001). Measurement of complex optical constants of a highly doped Si wafer using terahertz ellipsometry. Appl. Phys. Lett..

[43] Hofmann T (2010). Variable-wavelength frequency-domain terahertz ellipsometry. Rev. Sci. Instrum..

[44] Gopalan P (2020). The anisotropic quasi-static permittivity of single-crystal β-Ga_2_O_3_ measured by terahertz spectroscopy. Appl. Phys. Lett..

[45] Kim C, Ahn JS, Ji T, Eom JB (2017). Terahertz transmission properties of silicon wafers using continuous-wave terahertz spectroscopy. Meas. Sci. Technol..

[46] Nagashima T, Tani M, Hangyo M (2013). Polarization-sensitive THz-TDS and its application to anisotropy sensing. J. Infrared Millimeter Terahertz Waves.

[47] Neshat M, Armitage NP (2013). Developments in THz range ellipsometry. J. Infrared. Millimeter Terahertz Waves.

[48] Yatsugi K, Matsumoto N, Nagashima T, Hangyo M (2011). Transport properties of free carriers in semiconductors studied by terahertz time-domain magneto-optical ellipsometry. Appl. Phys. Lett..

[49] Hofmann T (2011). Terahertz ellipsometry and terahertz optical-Hall effect. Thin Solid Films.

[50] Kühne P (2018). Advanced terahertz frequency-domain ellipsometry instrumentation for in situ and ex situ applications. IEEE Trans. Terahertz Sci. Technol..

[51] Knight S (2017). In-situ terahertz optical Hall effect measurements of ambient effects on free charge carrier properties of epitaxial graphene. Sci. Rep..

[52] Schöche S (2011). Terahertz optical-Hall effect characterization of two-dimensional electron gas properties in AlGaN/GaN high electron mobility transistor structures. Appl. Phys. Lett..

[53] Hofmann T, Herzinger CM, Tiwald TE, Woollam JA, Schubert M (2009). Hole diffusion profile in a *p*-*p*^+^ silicon homojunction determined by terahertz and midinfrared spectroscopic ellipsometry. Appl. Phys. Lett..

[54] Han J, Zhu Z (2004). Terahertz frequency magneto-optical effect of GaN thin film. Surf. Sci..

[55] Kasic A, Schubert M, Einfeldt S, Hommel D, Tiwald TE (2000). Free-carrier and phonon properties of *n*- and *p*-type hexagonal GaN films measured by infrared ellipsometry. Phys. Rev. B.

[56] Yatsugi, K., Matsumoto, N., Nagashima, T. & Hangyo, M. Transport properties of free carriers in high quality n-type GaN wafers studied by THz time-domain magneto-optical ellipsometry. In *2011 Int. Conf. Infrared Millim. Terahertz Waves*, 1–2 10.1109/irmmw-THz.2011.6104886 (2011).

[57] Matsumoto N, Hosokura T, Nagashima T, Hangyo M (2011). Measurement of the dielectric constant of thin films by terahertz time-domain spectroscopic ellipsometry. Opt. Lett..

[58] Tachi K (2017). Measurement of the properties of GaN layers using terahertz time-domain spectroscopic ellipsometry. Phys. Status Solidi.

[59] Yamashita M, Takahashi H, Ouchi T, Otani C (2014). Ultra-broadband terahertz time-domain ellipsometric spectroscopy utilizing GaP and GaSe emitters and an epitaxial layer transferred photoconductive detector. Appl. Phys. Lett..

[60] Kawamura F (2003). Novel liquid phase epitaxy (LPE) growth method for growing large GaN single crystals: Introduction of the flux film coated-liquid phase epitaxy (FFC-LPE) method. Jpn. J. Appl. Phys..

[61] Imanishi M (2017). Homoepitaxial hydride vapor phase epitaxy growth on GaN wafers manufactured by the Na-flux method. Cryst. Growth Des..

[62] Imanishi M (2015). Dramatic reduction of dislocations on a GaN point seed crystal by coalescence of bunched steps during Na-flux growth. J. Cryst. Growth.

[63] Imanishi M (2019). Promotion of lateral growth of GaN crystals on point seeds by extraction of substrates from melt in the Na-flux method. Appl. Phys. Express.

[64] Hibberd MT (2016). Dielectric response of wurtzite gallium nitride in the terahertz frequency range. Solid State Commun..

